# *Trichinella spiralis* Thioredoxin Peroxidase 2 Regulates Protective Th2 Immune Response in Mice by Directly Inducing Alternatively Activated Macrophages

**DOI:** 10.3389/fimmu.2020.02015

**Published:** 2020-09-25

**Authors:** Qi-Wang Jin, Nian-Zhang Zhang, Wen-Hui Li, Hong-Tao Qin, Yin-Ju Liu, John Asekhaen Ohiolei, Dong-Yu Niu, Hong-Bin Yan, Li Li, Wan-Zhong Jia, Ming-Xin Song, Bao-Quan Fu

**Affiliations:** ^1^State Key Laboratory of Veterinary Etiological Biology, Key Laboratory of Veterinary Parasitology of Gansu Province, Lanzhou Veterinary Research Institute, Chinese Academy of Agricultural Sciences, Lanzhou, China; ^2^College of Veterinary Medicine, Northeast Agricultural University, Harbin, China; ^3^Jiangsu Co-innovation Center for Prevention and Control of Important Animal Infectious Diseases and Zoonoses, Yangzhou, China

**Keywords:** *Trichinella spiralis*, thioredoxin peroxidase-2, Th2 immune responses, macrophage, alternative activation

## Abstract

*Trichinella* infection can induce macrophages into the alternatively activated phenotype, which is primarily associated with the development of a polarized Th2 immune response. In the present study, we examined the immunomodulatory effect of *T. spiralis* thioredoxin peroxidase-2 (TsTPX2), a protein derived from *T. spiralis* ES products, in the regulation of Th2 response through direct activation of macrophages. The location of TsTPX2 was detected by immunohistochemistry and immunofluorescence analyses. The immune response *in vivo* induced by rTsTPX2 was characterized by analyzing the Th2 cytokines and Th1 cytokines in the peripheral blood. The rTsTPX2-activated macrophages (M_rTsTPX2_) were tested for polarization, their ability to evoke naïve CD4^+^ T cells, and resistance to the larval infection after adoptive transfer in BALB/c mice. The immunolocalization analysis showed TsTPX2 in cuticles and stichosome of *T. spiralis* ML. The immunostaining was detected in cuticles and stichosome of *T. spiralis* Ad3 and ML, as well as in tissue-dwellings around ML after the intestines and muscle tissues of infected mice were incubated with anti-rTsTPX2 antibody. Immunization of BALB/c mice with rTsTPX2 could induce a Th1-suppressing mixed immune response given the increased levels of Th2 cytokines (IL-4 and IL-10) production along with the decreased levels of Th1 cytokines (IFN-γ, IL-12, and TNF-α). *In vitro* studies showed that rTsTPX2 could directly drive RAW264.7 and peritoneal macrophages to the M2 phenotype. Moreover, M_rTsTPX2_ could promote CD4^+^ T cells polarized into Th2 type *in vitro*. Adoptive transfer of M_rTsTPX2_ into mice suppressed Th1 responses by enhancing Th2 responses and exhibited a 44.7% reduction in adult worm burden following challenge with *T. spiralis* infective larval, suggesting that the TsTPX2 is a potential vaccine candidate against trichinosis. Our study showed that TsTPX2 would be at least one of the molecules to switch macrophages into the M2 phenotype during *T. spiralis* infection, which provides a new therapeutic approach to various inflammatory disorders like allergies or autoimmune diseases.

## Introduction

*Trichinella spiralis* is a significant worldwide parasitic nematode that infects humans and other mammalian species, leading to trichinosis (1). Its life cycle involves three main stages-adults (Ad), newborn larvae (NBL), and muscle larvae (ML) (2). During the initial intestinal phase, *T. spiralis* elicits a Th1 type immune response (3). The Th2 type response is activated once the worm enters the enterocyte and becomes a well-characterized phenotype during the long-lasting infection of the muscles (4). The Th2 response to helminths is orchestrated by CD4^+^ T cells and depends mainly on highly elevated type 2 cytokines (interleukin-4, IL-5, etc.). The Th2 immune response accelerates the formation of niche or cystica, which prevents the immune system from killing the *T. spiralis*. On the other hand, it may dislodge the parasite and repair damage, thus protecting the host from excessive harm (5).

A macrophage is an important factor that regulates the immune system and has a central role in turning innate immune responses to adaptive responses (6). The macrophages display different functions based on the model of activation. Classically activated macrophages (CAMacs, also known as M1) are typically instructed by lipopolysaccharide (LPS) and interferon-γ (IFN-γ). In mice, M1 can produce intracellular-killing nitric oxide (iNO) and instigate Th1 biased responses in the host (7–9). In contrast, alternatively activated macrophages (AAMacs, also known as M2) are induced by IL-4 and IL-13. M2 macrophages abundantly express mannose receptor (MRC-1), arginase-1 (Arg-1), and chitinase-like protein (Chil3, Ym1) in mice, which activate the Th2 responses in the host (10–12). Throughout the life cycle of *T. spiralis*, excretory/secretory (ES) products are considered crucial compounds, which modulate macrophage function toward the alternative phenotype *in vitro* or *in vivo* (13, 14). However, it remains unclear whether these components derived from *T. spiralis* ES products regulate Th2 immune responses or whether these components are induced through direct action on macrophage or other immune cells.

Thioredoxin peroxidases (TPX) belong to a family of antioxidant enzymes characterized by 2-cys residues, which protect helminths from host reactive oxygen species (ROS) (15, 16). Our previous study revealed that three TPXs were expressed in three main stages of *T. spiralis* (17); the TPX2 gene expression levels were the highest in Ad3, and lowest in NBL, which matched well with the time of switching from Th1 to Th2 immune responses (17). The recombinant proteins were showed to remove exogenous H_2_O_2_ *in vitro* (17). TPXs from *Fasciola hepatica* can drive Th2 responses through a mechanism involving AAMacs (18, 19). Nevertheless, limited information is available on the ability of *T. spiralis* TPXs in regulating immune responses.

In the present study, we determined the polarization of macrophages that are modulated by the purified recombinant TsTPX2 (rTsTPX2) and the regulation of immune responses induced by the activated macrophages. After adoptive transfer of rTsTPX2-activated macrophages into BALB/c mice, we also evaluated the immune responses against *T. spiralis* infective muscle larval infection. Identification of the role of TsTPX2 in the regulation of immune responses during *T. spiralis* infection might help understand immunomodulatory mechanisms exploited by this parasite to create an environment suitable for its survival in the host organism.

## Materials and Methods

### Animals

Specific-pathogen-free (SPF) female Kunming mice and BALB/c mice, 6–8 weeks old, were purchased from Lanzhou Veterinary Research Institute Animal Center (Lanzhou, China). All animals were housed in an environment with a temperature of 22 ± 1°C, a relative humidity of 50 ± 1%, and a light/dark cycle of 12/12 h. All animal studies (including mice euthanasia procedure) were carried out in compliance with the regulations and guidelines of Lanzhou Veterinary Research Institute, Chinese Academy of Agricultural Sciences (Approval No. LVRIAEC2019-012), institutional animal care and conducted according to the Association for Assessment and Accreditation of Laboratory Animal Care (AAALAC) and the Institute of Animal Care and Use Committee (IACUC) guidelines.

### Parasites

The Chinese *T. spiralis* Henan strain (ISS534) was maintained in Kunming mice. The ML were isolated from infected mice *via* the conventional artificial digestion method, as previously described (20). The Ad3 were isolated from the small intestine of infected mice 3 days post-infection with ML *via* the standard approach, as previously shown (20).

### Recombinant Protein Preparation and Anti-rTsTPX2 Polyclonal Antibody Production

The rTsTPX2 was expressed through the prokaryotic expression system *E. coli* BL-21(DE3)/pET-30a (+) and purified as previously described (17). The endotoxin in the purified rTsTPX2 protein was removed by Pierce High-Capacity Endotoxin Removal Resin (Thermo Fisher Scientific, United States) according to the manufacturer’s protocol. The purified rTsTPX2 was used for functional analysis and immunization for preparing polyclonal antibody. Briefly, a 6-month-old rabbit was given a hypodermic injection of 500 μg of rTsTPX2 followed by three boosts immunization with 300 μg of rTsTPX2 at a 14-day interval. Seven days after the final boost, blood was collected by heart puncture, and sera were purified with affinity chromatography method.

### Immunofluorescence and Immunohistochemistry

*T. spiralis* ML harvested at 35 days post-infection were fixed by ice-cold methyl alcohol and incubated in Triton-100 overnight. Worms were then incubated with anti-rTsTPX2 antibodies and Alexa Fluor^®^ 488 labeled secondary antibodies. Stained *T. spiralis* ML were imaged with a confocal laser scanning microscope (Leica TCS SP8).

Small intestines harvested at 3 days post-infection and diaphragms harvested at 35 days post-infection from *T. spiralis* infected BALB/c mice were fixed in 4% paraformaldehyde. Thin sections of the embedded tissues were first stained with anti-rTsTPX2 antibodies and then incubated with Alexa Fluor^®^ 488 labeled secondary antibodies or DAB Quanto (Thermo Fisher Scientific). Confocal images of stained tissues were obtained with a confocal laser scanning microscope (Leica TCS SP8).

### Immunization of BALB/c Mice

Eight BALB/c mice per group were subcutaneously immunized with rTsTPX2 (50 μg per mouse) and boosted in the second week. Mice that received phosphate-buffered saline (PBS) and BSA were used as controls. The serum samples were collected at 3 weeks post the first injection. Anticoagulant blood samples were collected at 3 weeks post the first injection for Flow Cytometry analysis.

### Cell Isolation and Culture

The RAW 264.7 murine macrophages were obtained from the China Center for Type Culture Collection and were maintained in our laboratory. The cells were cultured in Dulbecco’s Modified Eagle’s Medium (DMEM, GIBCO) supplemented with 10% FCS (GIBCO), 2 mM L-glutamine (GIBCO) and 100 U/ml penicillin and 100 μg/ml streptomycin (GIBCO) at 37°C under 5% atmospheric CO_2_.

Resident peritoneal macrophages were obtained from peritoneal lavage, as previously reported (21, 22). Briefly, BALB/c mice were euthanized and sterilized. Mice were then placed in 75% ethanol for 5 s, after which, the peritoneum was exposed without injuring the peritoneal membrane. Six milliliters of ice-cold PBS were then injected into the peritoneal cavity. The mouse was shacked for 20 s to detach and suspend the macrophages. Consequently, the cell suspension was collected with a 21G needle in a 10 mL syringe. After repeating the process of cell collection, the cell suspension was transferred into a 50 mL tube containing 20 mL of ice-cold PBS and placed on ice. The cell suspension was then centrifuged and re-suspended in RPMI-1640 with 5% FCS (GIBCO), 2 mM L-glutamine (GIBCO), and 100 U/ml penicillin and 100 μg/ml streptomycin (GIBCO), and finally incubated in a 12-well cell-culture plate at 37°C for adherence. The non-adherences were removed every 2 h three times. The adherent cells were incubated at 37°C under 5% atmospheric CO_2_ overnight for further study.

Splenocytes were isolated from BALB/c mice, as previously described (23, 24). Mice were killed by exsanguination and placed in 75% ethanol for 5 s, after which the spleen was harvested and connective tissues were removed. The fragments of spleen were then gently pressed against the strainer with a syringe plunger. The cells were flushed through the strainer with 5 mL Mouse 1 × Lymphocyte Medium and transferred to a 15 mL tube. The cell suspension was then gently mixed with 1 mL RPMI 1640 medium and centrifuged at 800 × *g* for 30 min at room temperature. Consequently, the lymphocyte layer was collected, and cells were washed with RPMI1640 medium two times. Finally, the lymphocyte layer was re-suspended with RPMI1640 medium for further use.

CD4^+^ T cells were isolated from these splenocytes using anti-CD4 magnetic beads (Miltenyi Biotech) according to the manufacturer’s instructions. There were approximately 95% harvested CD4^+^ T cells after the FACS analysis.

### Activation of *in vitro* Macrophages

RAW264.7 or resident peritoneal macrophages were induced in the presence of 50 μg/ml of rTsTPX2 (RAW_tpx_ and M_rTsTPX2_), 10 ng/ml of IL-4 (RAW_IL–4_ and M_IL–4_) as M2 positive control, 10 ng/ml of IFN-γ (RAW_IFN–γ_ and M_IFN–γ_) as M1 positive control, 50 μg/ml of BSA as mimic control or PBS as no-treatment control (NTC). After 24 h cultivation at 37°C, cells were washed with PBS and then stored at −80°C for further study.

### Co-culture of Activated Macrophages and CD4^+^ T Cells

The activated macrophages were adjusted to 1 × 10^6^ cells/ml after washing with RPMI1640. CD4^+^ T cells collected from healthy BALB/c mice were re-suspended at the concentration of 5 × 10^6^ cells/ml. A total of 50 μl of activated macrophages was added to the CD4^+^ T cells with the same volume in 96-well plates at a humidified atmosphere of 5% CO_2_ at 37°C for 72 h. The culture supernatant was harvested for ELISA analysis of IL-4 and IFN-γ cytokines. The proliferation of CD4^+^ T cells was measured using the MTs kit (Promega), and the stimulation index was calculated according to the following formula: Proliferationindex=[(OD_protein_−OD_1640_)−(OD_PBS_−OD_1640_)]÷(OD_PBS_−OD_1640_).

### Adoptive Transfer of rTsTPX2-Activated Macrophages and Larvae Challenge in BALB/c Mice

A total of 5 × 10^5^ M_rTsTPX2_ re-suspended in 200 μl PBS was intraperitoneally injected into BALB/c mice. The peritoneal macrophages treated with IL-4 (M_IL–4_), IFN-γ (M_IFN–γ_), BSA (M_BSA_), or PBS (M_PBS_) were transferred into mice as controls. The serum and anticoagulated blood samples were collected at 3 weeks post-injection. At 3 weeks post-injection, mice from each group were orally challenged with 500 infective *T*. *spiralis* ML. The adults were determined at 3 days (Ad3) after infection. The reduction rates of Ad3 burden in activated-macrophages-transfer mice were evaluated according to the following formula: Worm reduction% = (1-mean number of worm in activated-macrophages-transfer mice/mean number of worm in unactivated-macrophages-transfer mice) × 100%.

### Cytokines Analysis

The levels of cytokines produced in the supernatant of activated macrophages co-cultured with CD4^+^ T cells (IL-4 and IFN-γ) and in mice serum samples (IL-4, IL-10, IL-12p70, TNF-α and IFN-γ) were measured by LEGEND MAX^TM^ Mouse ELISA kits (Biolegend) according to the manufacturer’s protocol. The sensitivity of detection was 0.5 pg/mL for IL-4, 2.7 pg/mL for IL-10, 0.5 pg/mL for IL-12p70, 1.5 pg/mL for TNF-α and 8 pg/mL for IFN-γ.

### Flow Cytometry Analysis of T Cells

The anticoagulated blood sample (100 μl) from each group was blocked with 0.4 μg anti-mouse CD16/32 antibody (Biolegend) for 15 min at room temperature (RT), followed by incubation with 0.2 μg fluorescent-labeled detection antibodies for 2 h at 4°C in the dark. T cells were stained with the following antibodies: PerCP-Cy5.5 Hamster anti-mouse CD3e (BD Biosciences), FITC Rat anti-mouse CD4 (BD Biosciences), PE Rat anti-mouse CD8a (BD Biosciences) and Isotype control antibodies (BD Biosciences). Erythrocytes in blood samples were lysed with 500 μl Red Blood Cell Lysis Buffer (Tiangen) on ice in the dark. The treated samples were then centrifuged at 500 × *g* for 10 min. Then, the pellet was re-suspended in 400 μl PBS. The suspensions were analyzed using a BD Accuri^TM^ C6 Plus flow cytometer (BD Biosciences). All data sets were analyzed with Flowjo software (TreeStar, Ashland, OR, United States).

### Quantitative Real-Time PCR Assays

Total RNA from macrophages was reverse-transcribed into cDNA using PrimeScript^TM^ RT reagent Kit with gDNA Eraser (TaKaRa Biotechnology). The qRT-PCR experiments were performed using TB Green^®^ Premix Ex Taq^TM^ II (Tli RNaseH Plus) kit (TaKaRa Biotechnology) on CFX96 Touch Real-Time PCR System (Bio-Rad). The qPCR primers were designed using Primer 3.0 online software^1^ for the following targets: GAPDH: 5′-AGGTCGGTGTGAACGGATTTG-3′ and 5′-TG TAGACCATGTAGTTGAGGTCA-3′; Arg-1: 5′-CTCCAAGCC AAAGTCCTTAGAG-3′ and 5′-AGGAGCTGTCATTAGGGA CATC-3′; MRC-1: 5′-CTCTGTTCAGCTATTGGACGC-3′ and 5′-CGGAATTTCTGGGATTCAGCTTC-3′; iNOS-1: 5′-ACAT TCAGATCCCGAAACGC-3′ and R: 5′-GACAATCCACAACTC GCTCC-3′; CCL22: 5′-AGGTCCCTATGGTGCCAATGT-3′ and R: 5′-CGGCAGGATTTTGAGGTCC-3′. The relative mRNA expression of target genes was calculated using the comparative Ct method, with the formula 2^–ΔΔCT^ (25).

### Western Blotting

Macrophage pellets were re-suspended in 30 μl of RIPA buffer (Beyotime, China). After incubation on ice for 1 h, the lysates were centrifuged at 12,000 rpm for 30 min at 4°C. The supernatant was mixed with sample buffer (Genscript, China) and incubated in boiling water for 10 min. The total cellular protein (30 μg/well) was separated by 10% SDS-PAGE. The separated proteins were transferred onto polyvinylidene difluoride membranes (PVDF, Millipore), which were then blocked overnight with 5% skim milk in Tris-buffered saline containing 0.1% Tween-20 (TBST). The membranes were respectively incubated with primary antibodies to detect Arg-1 (1: 1000 Cell Signaling Technology) and MRC-1 (1: 1000 Proteintech). The housekeeping gene encoding beta-actin (1: 5000 Thermo Fisher Scientific) was used as an internal control. Finally, the target protein bands were visualized by ECL substrate (Advansta, China), and images were collected by the ChemiDoc XRS^+^ system (Bio-Rad, Inc.).

### Statistical Analysis

Statistical analysis including student’s *t*-test or one-way ANOVA was performed by IBM SPSS Statistics 19 software (IBM, Inc.). All experiments were run in triplicate. Data are expressed as the mean ± SD from each experiment. A *P* < 0.05 was considered statistically significant.

## Results

### *T. spiralis* TPX2 Is Distributed in Tissue-Dwellings and Worm-Organs

**FIGURE 1:**
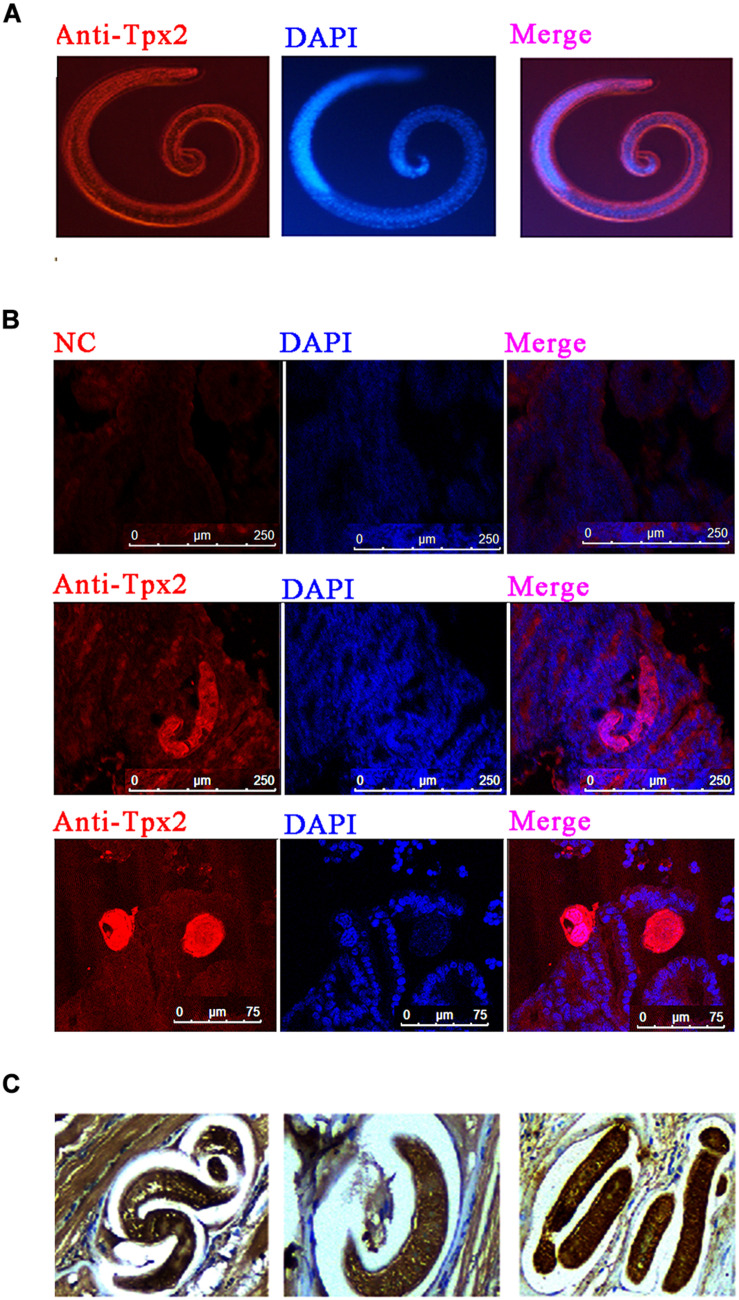
Immunolocalization of TPX2 at different *T. spiralis* phases. **(A)** Immunofluorescence of TsTPX2 in muscle larvae (ML) at 35 dpi. **(B)** Immunofluorescence of TPX2 in adult worms at 3 days. **(C)** Immunohistochemistry of TsTPX2 in ML at 35 dpi.

To determine the distribution of TPX2 in *T*. *spiralis*, immunofluorescence and immunohistochemistry tests were performed. The results of immunofluorescence on *T*. *spiralis* ML indicated that TPX2 appeared in cuticles and was sporadically expressed in the stichosome of ML (Figure 1A). The TPX2 was heavily expressed in cuticles and stichosome of Ad3 and was rarely presented in the surrounding (Figure 1B). In contrast, the immunohistochemistry results on the ML embedded diaphragm indicated that TPX2 was dense on both ML and the surroundings (Figure 1C). In our previous research, we found that the TPX2 gene was highly expressed in Ad3, while the expression levels were lowest in NBL (17) which matched well with the time of switching from Th1 to Th2 immune responses. Furthermore, our Western blot results showed that TsTPX2 is a significant component of *T*. *spiralis* ML ES product, which could react with sera from pigs infected by *T. spiralis* or from rabbit to produce anti-rTsTPX2 polyclonal antibody (Supplementary Figures 1, 2) (17). These data implied that TPX2 may play an essential role in switching Th1 to Th2 immune responses during *T*. *spiralis* infection.

### rTsTPX2 Induced a Th1-Suppressing Mixed Immune Response *in vivo*

**FIGURE 2:**
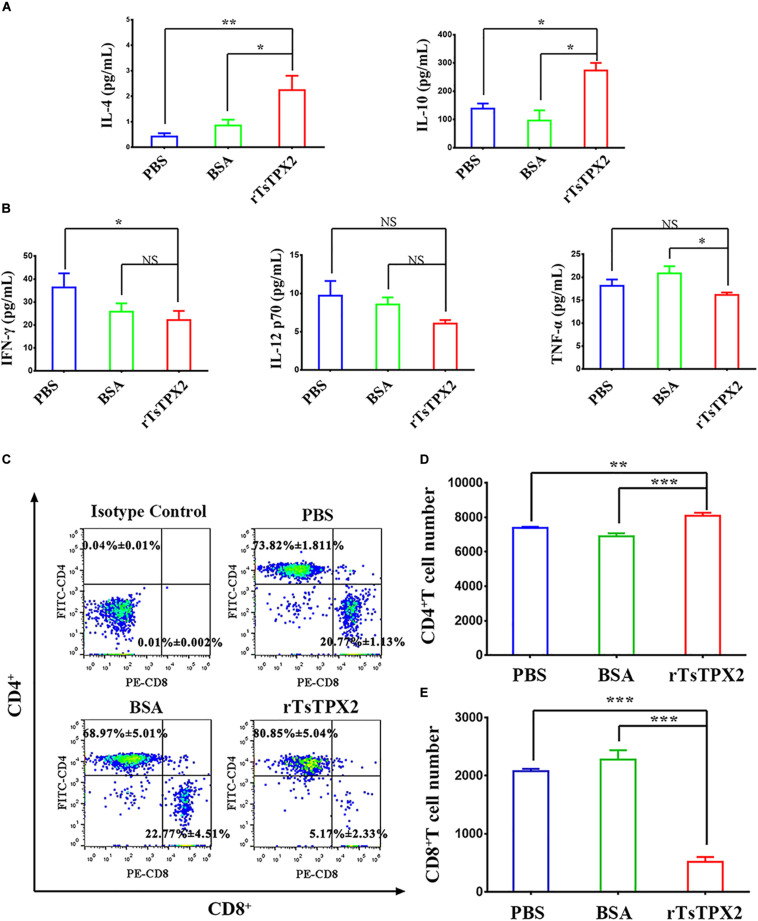
rTsTPX2 inhibits Th1 responses in mice. **(A)** The levels of Th2 cytokines in sera from mice detected by ELISA. **(B)** The levels of Th1 cytokines in sera from mice detected by ELISA. **(C)** Analysis of CD3^+^CD4^+^CD8^–^ and CD3^+^CD8^+^CD4^–^ T lymphocytes. The number on the representative contour plots showing the ratio of each subset out of peripheral lymphocytes. **(D)** The total number of CD3^+^CD4^+^CD8^–^ T cells in blood from mice. **(E)** The total number of CD3^+^CD8^+^CD4^–^ T cells in blood from mice. Experimental groups include mice challenged with PBS as a negative control, BSA as mock control, or rTsTPX2. Statistical analysis was performed with Student’s *t*-test, and data are mean ± SDs (representative of three experiments). ***P < 0.001, **0.001 < *P* < 0.01, *0.01 < *P* < 0.05. *n* >= 5 mice per group.

To determine the immunomodulatory effects of rTsTPX2, mice were subcutaneously immunized with the protein. At 3 weeks post-injection, the cytokines were examined in serum samples from mice by ELISA. The level of Th2 cytokines (IL-4 and IL-10) from mice immunized with rTsTPX2 was significantly higher than that in mice treated with BSA (*P* = 0.018 for IL-4 and *P* = 0.017 for IL-10) and PBS (*P* = 0.004 for IL-4 and *P* = 0.014 for IL-10) (Figure 2A). On the contrary, mice immunized with rTsTPX2 had a significantly lower level of Th1 cytokines (IFN-γ and TNF-α) compared to the control groups PBS (*P* = 0.018 for IFN-γ) and BSA (*P* = 0.044 for TNF-α). Moreover, no significant changes in the Th1 cytokine IL-12p70 were observed between mice immunized with rTsTPX2 and control groups (*P* = 0.137 for PBS and *P* = 0.073 for BSA), although a distinct trend of suppression was visible (Figure 2B). These results indicate that direct injection of rTsTPX2 could orchestrate the Th1 and Th2 programs in mice by promoting Th2 cytokines, IL-4 and IL-10, and simultaneously suppressing Th1 cytokines, IFN-γ, IL-12p70 and TNF-α. The results suggested that this protein could inhibit a type 1 immune response and is flexible in its ability to elicit a mixed Th1/Th2 response, which may be responsible for preventing excessive inflammation to create an environment suitable for *T. spiralis* survival in the host (26).

The anticoagulated blood samples were harvested to determine the cellular immune response by flow cytometry. Mice injected with rTsTPX2 showed more CD4^+^ T cells and fewer CD8^+^ T cells compared to controls (Figures 2C–E).

### rTsTPX2 Converts Macrophages to M2 Phenotype *in vitro*

**FIGURE 3:**
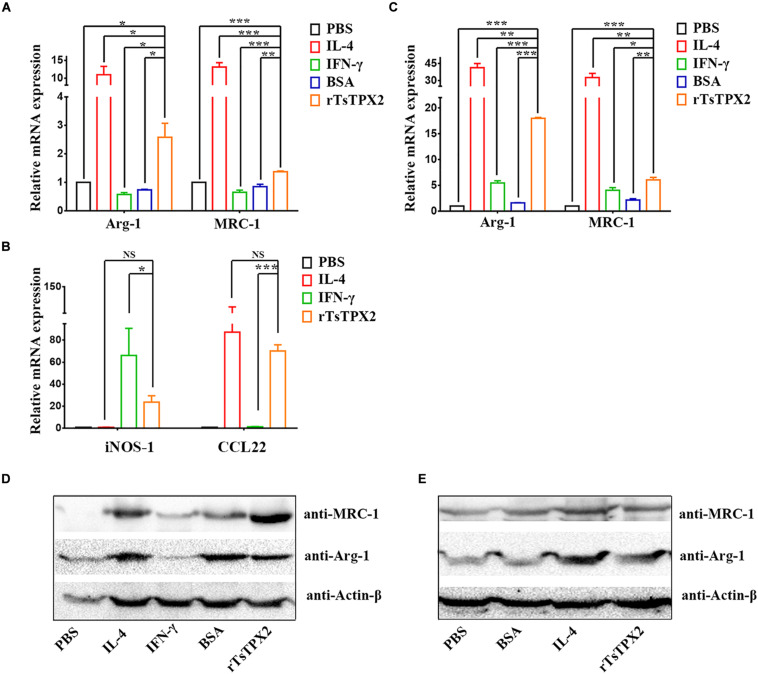
The phenotype of macrophages induced *in vitro.* **(A–E)** The expressions of M1 and M2 markers were analyzed by both qRT-PCR **(A–C)** and Western Blotting **(D,E)**. **(A,B)** Analysis of the expression of Arg-1 MRC-1, iNOS-1 and CCL22 genes in RAW264.7 co-cultured with various stimuli by qPCR. **(C)** Analysis of the expression of Arg-1 and MRC-1 genes in peritoneal macrophages isolated from healthy BALB/c mice after co-cultured with various stimuli by qPCR. **(D,E)** Analysis of the expression of Arg-1 and MRC-1 genes in RAW264.7 and peritoneal macrophages after co-cultured with various stimuli by Western Blotting. Experimental groups include macrophages stimulated with PBS as blank control, IL-4 as M2 positive control, INF-γ as M1 positive control, BSA as mock control or rTsTPX2. Statistical analysis was performed with Student’s *t*-test, and data are presented as mean ± SDs (representative of three experiments). ***P < 0.001, **0.001 < *P* < 0.01, *0.01 < *P* < 0.05. *n* >= 5 mice per group.

Given the presence of rTsTPX2 could suppress the Th1 response *in vivo*, we sought to exploit whether the protein triggers the regulation of host immune response by driving macrophages into the M2 phenotype (27). The peritoneal macrophages from BALB/c mice and commercial RAW264.7 were respectively stimulated with rTsTPX2 for 24 h *in vitro*. Arg-1 and MRC-1 gene expressions were examined by both qRT-PCR and Western blotting. The results of qRT-PCR showed that the expression of both Arg-1 and MRC-1 genes was significantly higher in RAW264.7 macrophages stimulated with rTsTPX2 than in those stimulated with IFN-γ (*P* = 0.0158 and 0.0008) or those stimulated with BSA (*P* = 0.0202 and 0.004), but was lower than those stimulated with IL-4 (*P* = 0.0245 and 0.0006) (Figure 3A). The stimulus of peritoneal macrophages from BALB/c mice was consistent with the results of RAW264.7 macrophages using qRT-PCR detection (Figure 3C). For the IFN-γ stimulus, the expression of Arg-1 and MRC-1 in peritoneal macrophages was slightly up-regulated when compared with the mock groups (PBS and BSA stimulus), but the difference was not significant (*P* > 0.05).

The Western blotting further indicated consistent results with qRT-PCR; Arg-1 and MRC-1 genes were significantly up-regulated in both RAW264.7 macrophages (Figure 3D), and peritoneal macrophages from BALB/c mice (Figure 3E) stimulated with rTsTPX2. These results above indicated that rTsTPX2 could drive macrophages to M2 phenotype *in vitro.*

Then the identification was further confirmed by detection of iNOS-1 and CCL22 gene expression in RAW264.7 cells using qRT-PCR. The results showed that there were no significant differences between RAW_tpx_ and RAW_IL–4_ groups for the expression of iNOS-1 (M1) and CCL22 (M2), which indicated that rTsTPX2 can induce macrophages to an M2 phenotype (Figure 3B).

### M_rTsTPX2_ Promote the Production of Th2 Cytokines From Naïve CD4^+^ T Cells *in vitro*

**FIGURE 4:**
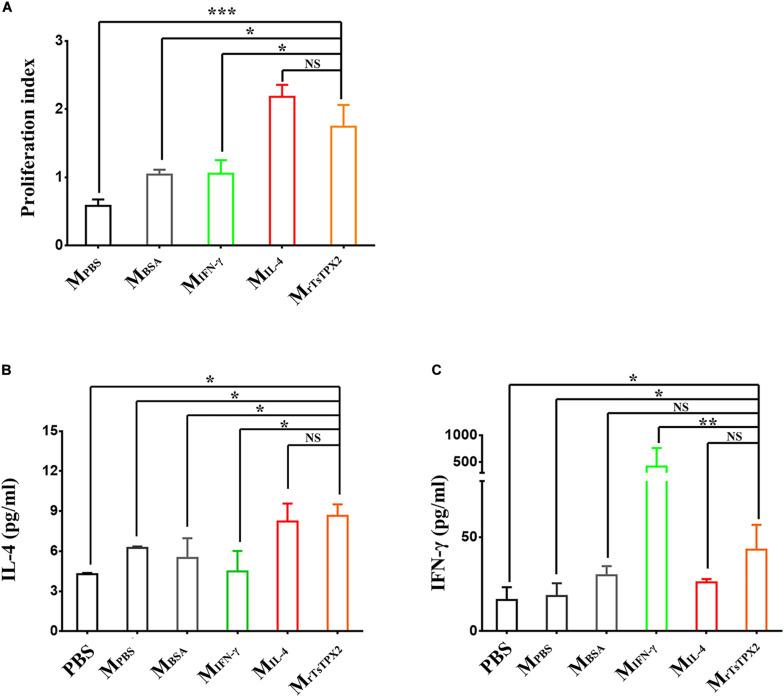
The differentiation and proliferation of CD4^+^ T cells induced by macrophages. **(A)** The proliferation index of CD4^+^ T cells induced by macrophages at 72 h after co-culture, measured by MTs kit. **(B)** Levels of IL-4 production in supernatant of CD4^+^ T cells co-cultured with macrophages. **(C)** Levels of IFN-γ production in supernatant of CD4^+^ T cells co-cultured with macrophages. CD4^+^ T cells isolated from healthy BALB/c mice co-cultured with macrophages induced by various stimuli, including PBS as blank control (M_PBS_), IL-4 as M2 positive control (M_IL–4_), INF-γ as M1 positive control (M_INF–γ_), BSA as mock control (M_BSA_) or rTsTPX2 (M_rTsTPX2_). The experimental group PBS representative CD4^+^ T cells dealing with PBS. Statistical analysis was performed with Student’s *t*-test, and data are presented as mean ± SDs (representative of three experiments). ***P < 0.001, **0.001 < *P* < 0.01, *0.01 < *P* < 0.05, NS, not significant. n >= 3 mice per group.

The observations that Th2 cytokines (IL-4 and IL-10) were significantly elevated upon administration of rTsTPX2 (Figure 2A) and that TPX could directly and alternatively activate macrophages (18), led us to evaluate whether the Th2 type immune response could be driven by M_rTsTPX2_. To determine the relationship between M_rTsTPX2_ and Th2 type immune response, we first examined the ability of M_rTsTPX2_ to induce proliferation of CD4^+^ T cells *in vitro*. As shown in Figure 4A, significantly higher proliferation of CD4^+^ T cells was observed after co-incubation with M_rTsTPX2_ than with M_IFN–γ_ (*P* = 0.015), M_BSA_ (*P* = 0.0305) or M_PBS_ (*P* = 0.0007); yet, no significant difference was found compared with co-inoculation with M_IL–4_ (*P* = 0.328).

ELISA assay was then used to analyze the cytokine production from the supernatant of the co-culture medium. When cultured with M_rTsTPX2_, the CD4^+^ T cells produced a compound of Th2 (IL-4) and Th1 (IFN-γ) cytokines compared to controls. A significantly higher production of IL-4 was detected in the supernatant of CD4^+^ T cells co-cultured with M_rTsTPX2_ compared to cells co-cultured with M_IFN–γ_ (*P* = 0.013), M_BSA_ (*P* = 0.05) or PBS (*P* = 0.01) (Figure 4B). Whereas the production of IFN-γ was reduced in the supernatant of CD4^+^ T cells co-cultured with M_rTsTPX2_ compared to those co-cultured with M_IFN–γ_ (*P* = 0.003) but was increased compared to cells co-cultured with M_PBS_ (*P* = 0.045) or PBS (*P* = 0.0356) (Figure 4C). The results revealed that M_rTsTPX2_ could elicit CD4^+^ T cell proliferation to promote a higher level of IL-4 production, indicating that a Th2 phenotype of CD4^+^ T cells were directly driven by the M_rTsTPX2_.

### Adoptive Transfer of M_rTsTPX2_ Weakens the Th1 Responses and Enhances the Th2 Immune Response in BALB/c Mice

**FIGURE 5:**
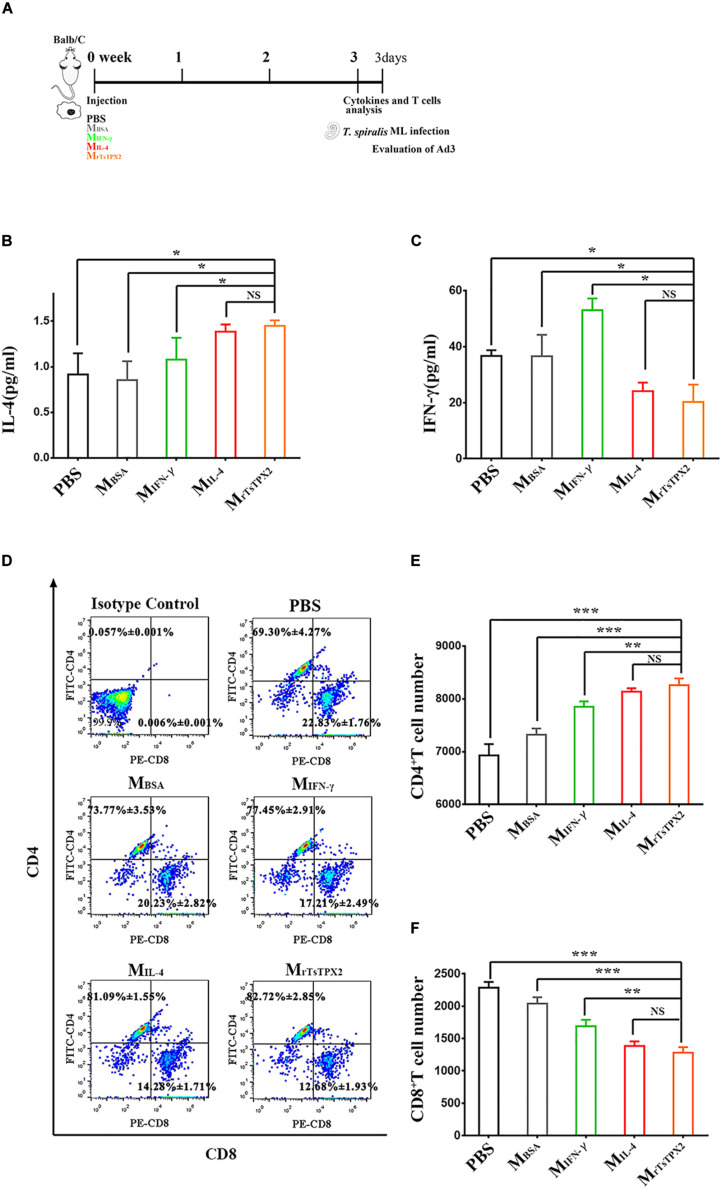
The type of immune responses in mice adoptively transferred with macrophages. **(A)** Schematics of immune responses in mice adoptively transferred with one of the macrophage types. Three weeks after the first injection, the immunological status was analyzed, and mice were experimentally infected with 500 *T. spiralis* ML. Analysis of adult load at 3 days after infection. **(B)** Levels of IL-4 production in sera from mice transferred with various macrophage types. **(C)** Levels of IFN-γ production in sera from mice transferred with various macrophage types. **(D)** Analysis of CD3^+^CD4^+^CD8^–^ and CD3^+^CD8^+^CD4^–^ T lymphocytes. The number on the representative contour plots showing the ratio of each subset out of peripheral lymphocytes. **(E)** The total number of CD3^+^CD4^+^CD8^–^ T cells in blood from mice. **(F)** The total number of CD3^+^CD8^+^CD4^–^ T cells in blood from mice. Experimental groups include mice transferred with macrophages activation by IL-4 as M2 positive control (M_IL–4_), INF-γ as M1 positive control (M_INF–γ_), BSA as mock control (M_BSA_) or rTsTPX2 (M_rTsTPX2_). The experimental group, PBS representative mice, injected with PBS. Statistical analysis was performed with Student’s *t*-test, and data are presented mean ± SDs (representative of three experiments). ***P < 0.001, **0.001 < *P* < 0.01, *0.01 < *P* < 0.05, NS, not significant. n >= 3 mice per group.

Whether M_rTsTPX2_ drives the Th2 type immune response was further evaluated by adoptively transferring M_rTsTPX2_ into healthy BALB/c mice through intraperitoneal injection. At 3 weeks after injection, we examined the cytokines and T subclasses in the peripheral blood, Figure 5A shows the experimental protocol of macrophage adoptive transfer. The level of IL-4 cytokine in mice transferred with M_rTsTPX2_ was significantly higher compared with those treated with PBS (*P* = 0.009), M_BSA_ (*P* = 0.015), or M_IFN–γ_ (*P* = 0.05). However, no significant difference in IL-4 production was detected in mice transferred with M_rTsTPX2_ and M_IL–4_ (*P* = 0.325) (Figure 5B). Regarding IFN-γ analysis, mice transferred with M_rTsTPX2_ were significantly lower than those injected with PBS (*P* = 0.035), M_BSA_ (*P* = 0.05) or M_IFN–γ_ (*P* = 0.01), and the difference between M_rTsTPX2_ and M_IL–4_ transferred mice was not significant (*P* = 0.6) (Figure 5C). An injection of M_rTsTPX2_ into mice induced an increased IL-4 and a decreased IFN-γ, which is similar to results observed in mice transferred with M_IL–4_. These results demonstrated that the rTsTPX2-activated macrophages could elicit a Th2 immune response in mice.

Mice transferred with M_rTsTPX2_ had elevated CD4^+^ T cell percentage and reduced CD8^+^ T cell percentage compared to the control groups (Figure 5D). The count of CD4^+^ T cells in mice transferred with M_rTsTPX2_ were significantly higher compared to the PBS (*P* = 0.0004), M_BSA_ (*P* = 0.0002) and M_IFN–γ_ (*P* = 0.0270) groups (Figure 5E). No significant difference in CD4^+^ T cells was detected in mice transferred with M_rTsTPX2_ and M_IL–4_ (*P* = 0.3790) (Figure 5E). In contrast, the CD8^+^ T cell numbers in mice transferred with M_rTsTPX2_ were significantly lower than those injected with PBS (*P* < 0.0001), M_BSA_ (*P* = 0.00013) and M_IFN–γ_ (*P* = 0.0105). The difference of CD8^+^ T cell numbers between mice transferred with M_rTsTPX2_ and M_IL–4_ was not statistically significant (*P* = 0.3720) (Figure 5F). The increased CD4^+^ T cells along with the decreased CD8^+^ T cells in mice transferred with M_rTsTPX2_ implied that the rTsTPX2 induced macrophages could provide protection as the Th2 cells mediated the expulsion of worms from the mice.

### Adoptive Transfer of M_rTsTPX2_ Induced Protective Immunity

**FIGURE 6:**
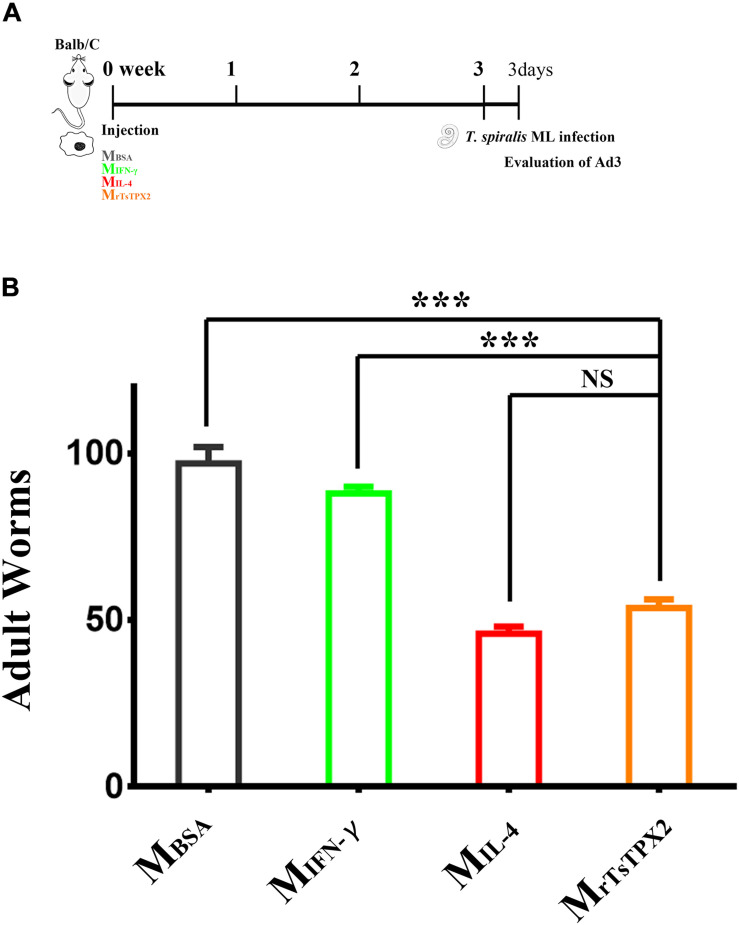
Numbers of 3-day adults from the macrophages transferred mice. **(A)** Schematics of immune responses in mice adoptively transferred with one of the macrophage types. Three weeks after the first injection, the immunological status was analyzed, and mice were experimentally infected with 500 *T. spiralis* ML. Analysis of adult load at 3 days after infection. **(B)** Adult load in the intestine of mice. Experimental groups include mice transferred with macrophages activation by IL-4 as M2 positive control (M_IL–4_), INF-γ as M1 positive control (M_INF–γ_), BSA as mock control (M_BSA_) or rTsTPX2 (M_rTsTPX2_). Statistical analysis was performed with Student’s *t*-test, and data presented are mean ± SDs (representative of three experiments). ***P < 0.001, NS: not significant. n >= 3 mice per group.

Macrophages activated by alternative pathways are considered to be involved in helminth containment. To determine whether M_rTsTPX2_ can protect mice from *T. spirali*s infection, mice were orally challenged with 500 infective larvae, followed by the adoptive transfer of M_rTsTPX2_. The adult worm burdens were evaluated at 3 days after infection. As shown in Figure 6, mice transferred with M_rTsTPX2_ had significantly lower numbers of Ad3 than those from M_BSA_ (*P* < 0.0001) and M_IFN–γ_ (*P* < 0.0001) groups. The Ad3 number in mice injected with M_rTsTPX2_ had a reduction of 44.7% compared to the M_BSA_ group. However, there was no significant difference of Ad3 loadings in mice injected with M_rTsTPX2_ and M_IL–4_ (*P* = 0.1240).

## Discussion

During the enteric phase, *T. spiralis* infection induces a Th1/Th2 mixed response, while in the systemic phase, the response is Th2-biased (28). During the transformation of immune responses, excretory-secretory (ES) products from the *T. spiralis* have been reported to play a crucial role in inhibiting inflammation (29) which can protect the parasite and the host at the same time (18, 19, 30). So far, only a structural molecule, chitin, has been identified to modulate the macrophage to the alternative phenotype (4, 31). The molecules identified in *T. spiralis* ES products that function in regulating the types of immune responses are still poorly understood when compared to other medical and veterinary important helminth species. In our previous study, the expression of TsTPX2 was up-regulated in both Ad3 and ML stages compared to NBL, almost matching the transformation of Th2 immune responses during *T. spiralis* infection (17). In addition, the TsTPX2 was detected in ML ES products by Western blotting analysis (Supplementary Figure 2) and in tissue-dwellings by immunohistochemistry and immunofluorescence techniques (Figure 1). Based on these results, we speculated that TsTPX2, as one of the ES products, might participate in the regulation of responses during *T. spiralis* infection. We found that rTsTPX2 could suppress Th1 immune responses by promoting Th2 cytokines, IL-4 and IL-10 and simultaneously demoting Th1 cytokines, IFN-γ, IL-12p70 and TNF-α. The ability of rTsTPX2 to inhibit a type 1 immune response is consistent with observations made in thioredoxin peroxidases derived from *Fasciola hepatica* and *Schistosoma mansoni* (18, 19).

Macrophages orchestrate Th1/Th2 responses by responding to different environmental signals (6). Exposure to IL-4 and IL-13 polarizes macrophages into the M2 phenotype and drives the host to the Th2 response (10–12, 32, 33). Here, we showed that rTsTPX2 could directly convert macrophages to an M2 phenotype *in vitro* (rTsTPX2 up-regulated Arg-1, CCL22 and MRC-1, as shown in Figure 3). Whereas, the transcription of Arg-1 and MRC-1 genes was also slightly up-regulated in IFN-γ stimulated cells (Figure 3C). The seeming paradox was eliminated through a sharp increase in expression of CAMs marker (iNOS-1 gene) in macrophages stimulated with IFN-γ compared to the M_IL–4_, M_rTsTPX2_, and mock control groups (Figure 3B and Supplementary Figure 4). The conversion of macrophages into an M2 phenotype by rTsTPX2 was further determined through proliferating naïve CD4^+^ T cells to secrete Th2 cytokine IL-4.

Naïve CD4^+^ T cells, the precursors of Th cells, undergo clonal expansion and differentiation into distinct effector Th cell subsets, and these Th cell subsets directly promote the control of pathogens by producing signature cytokines (5, 34, 35). Many studies have shown that CD4^+^ Th2 cells have a vital role in anti-helminth responses. The CD4^+^ T-cell-depleted mice failed to mount protective immune responses against *S. mansoni* after vaccination (35). Moreover, the *Nippostrongylus brasiliensis* expelling rate was significantly decreased in CD4^+^ T-cell-exhausted mice compared to normal controls (36); this capacity was recovered once CD4^+^ T cells were injected in the depleted mice (37). In this study, we found that CD4^+^ T cell proliferation occurred through rTsTPX2-activated macrophages *in vivo* and *in vitro*, in turn inducing the IL-4 production. Also, after co-culturing M_rTsTPX2_ with CD4^+^ T cells isolated from *T. spiralis* infected mice, the proliferation sharply increased (Supplementary Figure 3) suggesting that the rTsTPX2-activated macrophages could induce the memory of Th2 cells proliferation under the same situation.

The Th2 immune response has a critical role in anti-helminth immunity. The Th2 response is beneficial for the host not only to expel *T. spiralis* adults out of the intestines but also to repair or prevent muscle tissue damage (38, 39). Adoptive transfer of M_rTsTPX2_ into mice evoked an increase in IL-4 production along with decreased levels of IFN-γ indicating that the Th2 immune response was altered which is consistent with the observations from mice adoptively transferred with TsES-activated macrophage (30). The Th2 immune response in mice transferred with M_rTsTPX2_ was able to induce protection against *T. spiralis* demonstrating a 44.7% reduction in adult worms compared to the mimic control. The adult worms of *T. spiralis* release offspring to induce systemic infection and the reduction of adult worm reflects the effect of rTsTPX2 induced protective immunity against the worm infection (40).

During *T. spiralis* infection, the host’s immune response switched from Th1 to Th2 response. Our data indicated that regarding TsTPX2, at least one molecule has a significant role in modulating these immune transformations by inducing macrophages to an M2 phenotype. These immunomodulatory mechanisms were exploited by this parasite to create an environment suitable for its survival in the host organism. On the other hand, these mechanisms are important in establishing new therapeutic approaches for various inflammatory disorders like allergies or autoimmune diseases.

The humoral immunity also exhibits an important role against the helminth infection. IgG1 isotype shows potent anti-inflammatory activity. Meanwhile, the IgG production driven by worms can also participate in restricting extreme inflammatory responses during the chronic infection process (41–43). However, in the present study, we only evaluated the protective Th2 responses in cytokines secretion instead of antibody production. The efficacy of rTsTPX2 in inducing the humoral response should be further warranted in the next study.

## Conclusion

Our data suggested that TsTPX2 may directly induce macrophages to an M2 phenotype *in vitro* and *in vivo.* Immunization of mice with rTsTPX2 or M_rTsTPX2_ could increase the number of CD4 + T cells and protect against *T. spiralis* infection by mediating worms expulsion from the host. Importantly, understanding the ability of TsTPX2 in the regulation of macrophages into the M2 phenotype can not only provide us a new insight into immunomodulatory mechanisms exploited by *T. spiralis* to create an environment proper for its survival in the hosts, but also establish novel therapeutic methods to various inflammatory disorders like allergies or autoimmune diseases.

## Data Availability Statement

All datasets presented in this study are included in the article/Supplementary Material.

## Ethics Statement

The animal study was reviewed and approved by Animal Ethics Committee of Lanzhou Veterinary Research Institute, Chinese Academy of Agricultural Sciences.

## Author Contributions

B-QF, M-XS, and N-ZZ conceived the project, designed the experiments, and critically revised the manuscript. Q-WJ, W-HL, Y-JL, D-YN, and H-TQ performed the experiments and analyzed the data. Q-WJ, N-ZZ, and JO drafted and revised the manuscript. H-BY, LL, and W-ZJ helped in the implementation of the study. All authors reviewed and approved the final version of the manuscript.

## Conflict of Interest

The authors declare that the research was conducted in the absence of any commercial or financial relationships that could be construed as a potential conflict of interest.

## Footnotes

1 http://bioinfo.ut.ee/primer3-0.4.0/primer3/

## Supplementary Material

Supplementary Figure 1 Expression and purification of recombinant TsTPX2 in *E. coli*. **(A)** SDS-PAGE analysis the expression of recombinant TsTPX2. Lane 1: IPTG-induced inclusion body; Lane 2: IPTG-induced supernatant; Lane 3: renatured inclusion body; Lane 4: Purified renatured protein. Lane M: protein molecular weight marker. **(B)** Detection of rTsTPX2 by Western Blotting: Lane 1: rTsTPX2 reacted with anti-rTsTPX2 rabbit sera (1: 3200); Lane M: Protein molecular weight markers.

Supplementary Figure 2 Detection TsTPX2 from ML ES products. **(A)** SDS-PAGE analysis the ML ES products. **(B)** Detection of TsTPX2 from ML ES production by Western Blotting with an anti-rTsTPX2 polyclonal antibody. Lane 1: ML ES products; Lane M: Protein molecular weight markers. The ML ES samples were harvested from *T. spiralis* strain (ISS534) maintained in Kunming mice at 35 days after infection. The ES protein from ML (6 μg/well) was separated by 10% SDS-PAGE. The separated proteins were stained with CBB (Coomassie brilliant blue) reagents **(A)** or transferred onto polyvinylidene difluoride membranes (PVDF, Millipore), which were then blocked overnight with 5% skim milk in Tris-buffered saline containing 0.1% Tween-20 (TBST). The membranes were respectively incubated with primary antibody (anti-rTsTPX2 rabbit sera, 1:3200) and secondary antibody (goat anti-rabbit IgG (H + L) proteintech, 1: 5000).

Supplementary Figure 3 The proliferation of activated CD4^+^ T cells induced by macrophages. The CD4^+^ T cells were isolated from *T. spiralis* infected mice at 35 days after ML infection. The proliferation index of CD4^+^ T cells induced by macrophages at 72 h after co-culture measured by MTs kit. Statistical analysis was performed with Student’s *t*-test, and data are expressed as mean ± SDs (representative of three experiments). ****P* < 0.001, **0.001 < *P* < 0.01, *0.01 < *P* < 0.05, NS, not significant. *n* > = 3 mice per group.

Supplementary Figure 4 The phenotype of macrophages induced *in vitro* by flow cytometry. The expressions of MRC-1 **(A)** and iNOS-1 **(B)**. Genes were analyzed by flow cytometry; the left panel was determined by Flowjo software (TreeStar, Ashland, OR, United States), while the right panel shows the mean of fluorescence intensity experimental group. RAW264.7 macrophages were incubated with respective proteins for 48 h. The PBS was used as non-treated control; the recombined mouse Cyclic GMP-AMP synthase (rmcGAS) expressed in the same system with rTsTPX2 was used as irrelevant control; the recombined IL-4 as M2 positive control and IFN-γ as M1 positive control. The following fluorescence conjunct antibodies, Alexa Fluor^®^ 647 rat anti-mouse CD206 (MRC-1), FITC mouse anti-iNOS-1, PE rat anti-mouse F4/80, and the Isotype control antibodies were purchased from BD Biosciences (United States). After staining, the macrophages were firstly gated with F4/80-positive cells, then the iNOS-1 and CD206 (MRC-1) positive cells analyzed with mean of fluorescence intensity (MFI).
