# Supplementary figures and images for: Trichinella spiralis Thioredoxin Peroxidase 2 Regulates Protective Th2 Immune Response in Mice by Directly Inducing Alternatively Activated Macrophages
Source: Front Immunol. 2020 Sep 25;11:2015. doi: 10.3389/fimmu.2020.02015 (PMC7544948; doi:10.3389/fimmu.2020.02015)

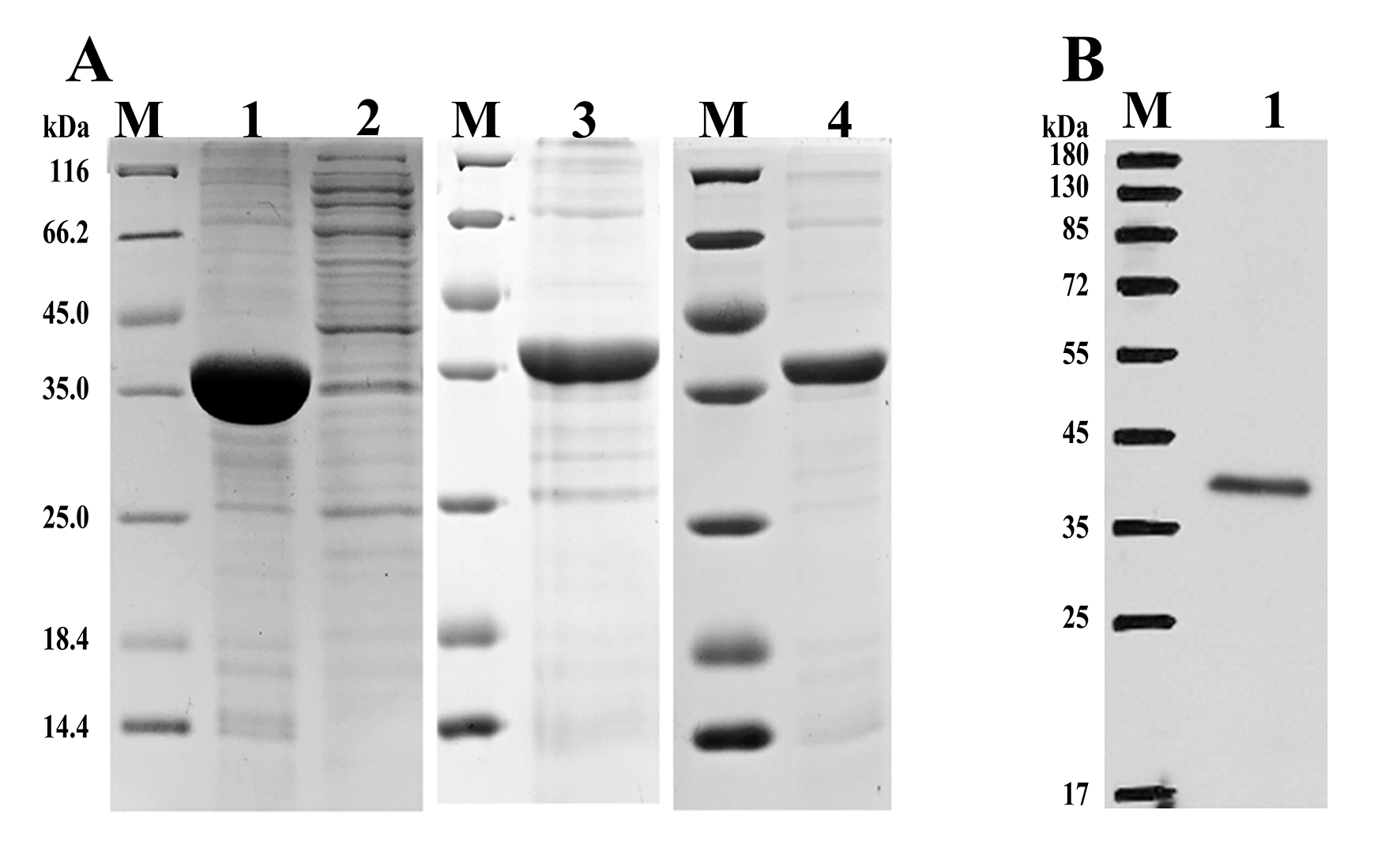

Supplement: Supplementary Figure 1 — Expression and purification of recombinant TsTPX2 in E. coli. (A) SDS-PAGE analysis the expression of recombinant TsTPX2. Lane 1: IPTG-induced inclusion body; Lane 2: IPTG-induced supernatant; Lane 3: renatured inclusion body; Lane 4: Purified renatured protein. Lane M: protein molecular weight marker. (B) Detection of rTsTPX2 by Western Blotting: Lane 1: rTsTPX2 reacted with anti-rTsTPX2 rabbit sera (1: 3200); Lane M: Protein molecular weight markers. [file Image_1.TIF]

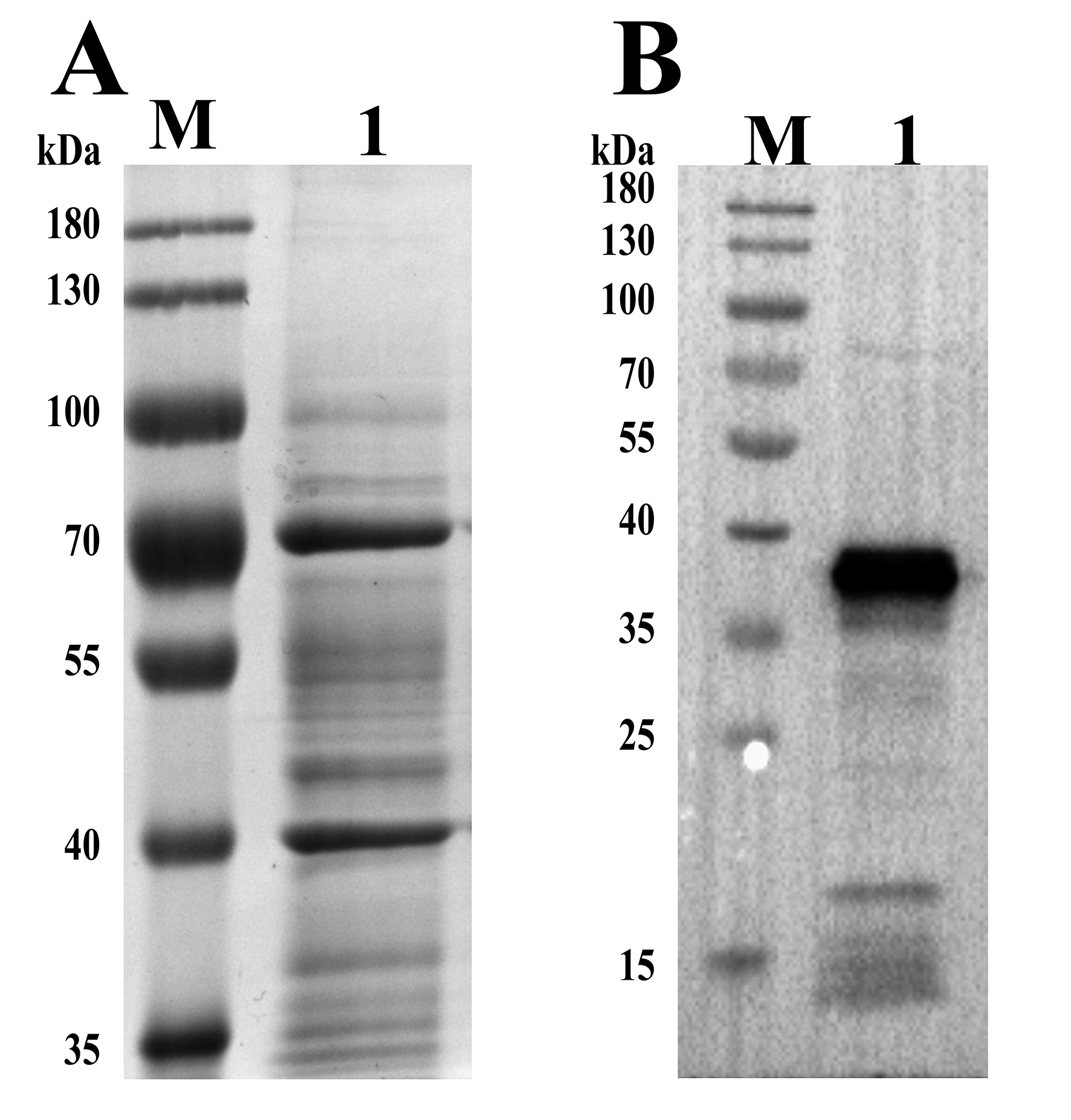

Supplement: Supplementary Figure 2 — Detection TsTPX2 from ML ES products. (A) SDS-PAGE analysis the ML ES products. (B) Detection of TsTPX2 from ML ES production by Western Blotting with an anti-rTsTPX2 polyclonal antibody. Lane 1: ML ES products; Lane M: Protein molecular weight markers. The ML ES samples were harvested from T. spiralis strain (ISS534) maintained in Kunming mice at 35 days after infection. The ES protein from ML (6 μg/well) was separated by 10% SDS-PAGE. The separated proteins were stained with CBB (Coomassie brilliant blue) reagents (A) or transferred onto polyvinylidene difluoride membranes (PVDF, Millipore), which were then blocked overnight with 5% skim milk in Tris-buffered saline containing 0.1% Tween-20 (TBST). The membranes were respectively incubated with primary antibody (anti-rTsTPX2 rabbit sera, 1:3200) and secondary antibody (goat anti-rabbit IgG (H + L) proteintech, 1: 5000). [file Image_2.TIF]

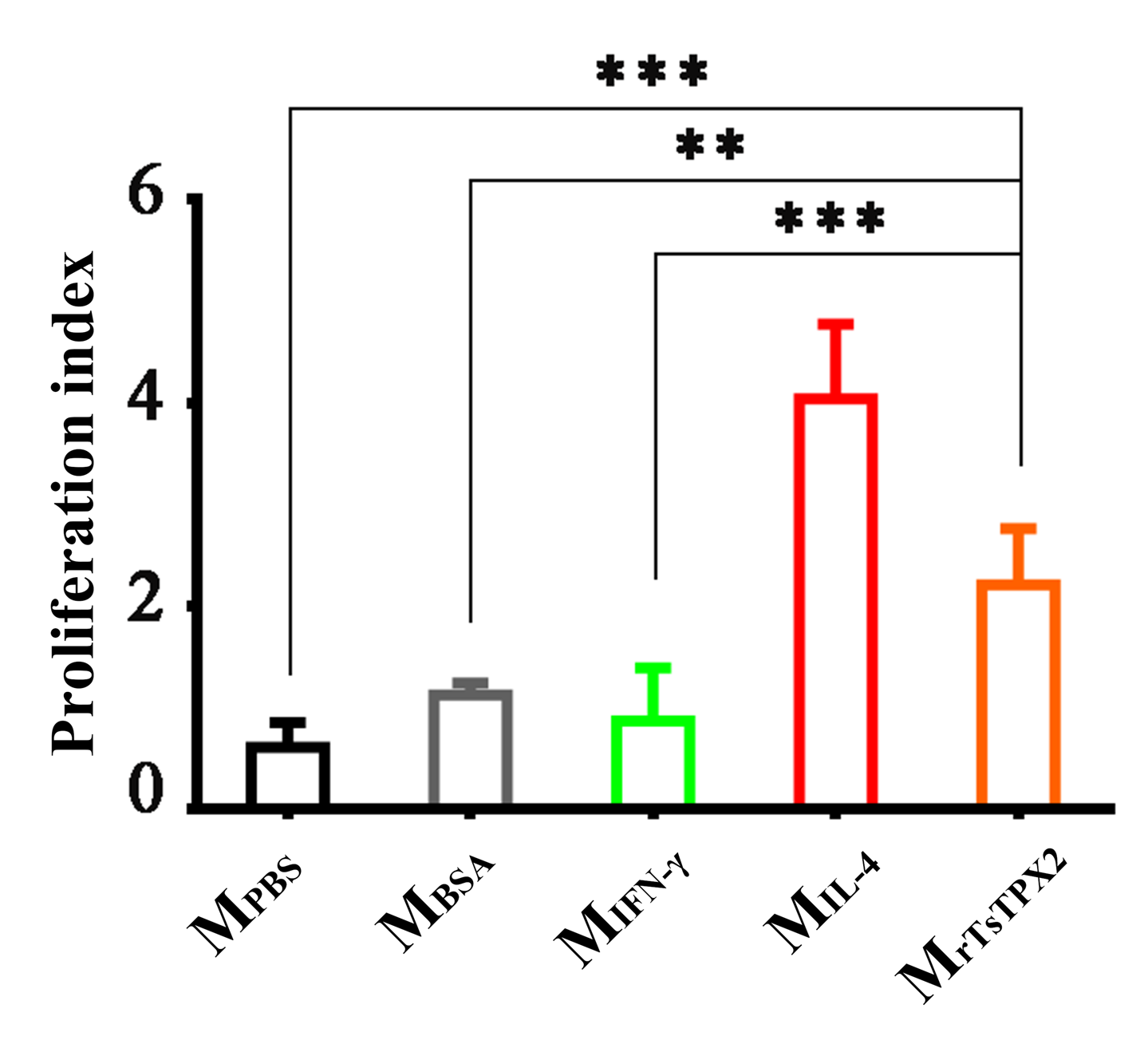

Supplement: Supplementary Figure 3 — The proliferation of activated CD4+ T cells induced by macrophages. The CD4+ T cells were isolated from T. spiralis infected mice at 35 days after ML infection. The proliferation index of CD4+ T cells induced by macrophages at 72 h after co-culture measured by MTs kit. Statistical analysis was performed with Student’s t-test, and data are expressed as mean ± SDs (representative of three experiments). ***P < 0.001, **0.001 < P < 0.01, *0.01 < P < 0.05, NS, not significant. n > = 3 mice per group. [file Image_3.TIF]

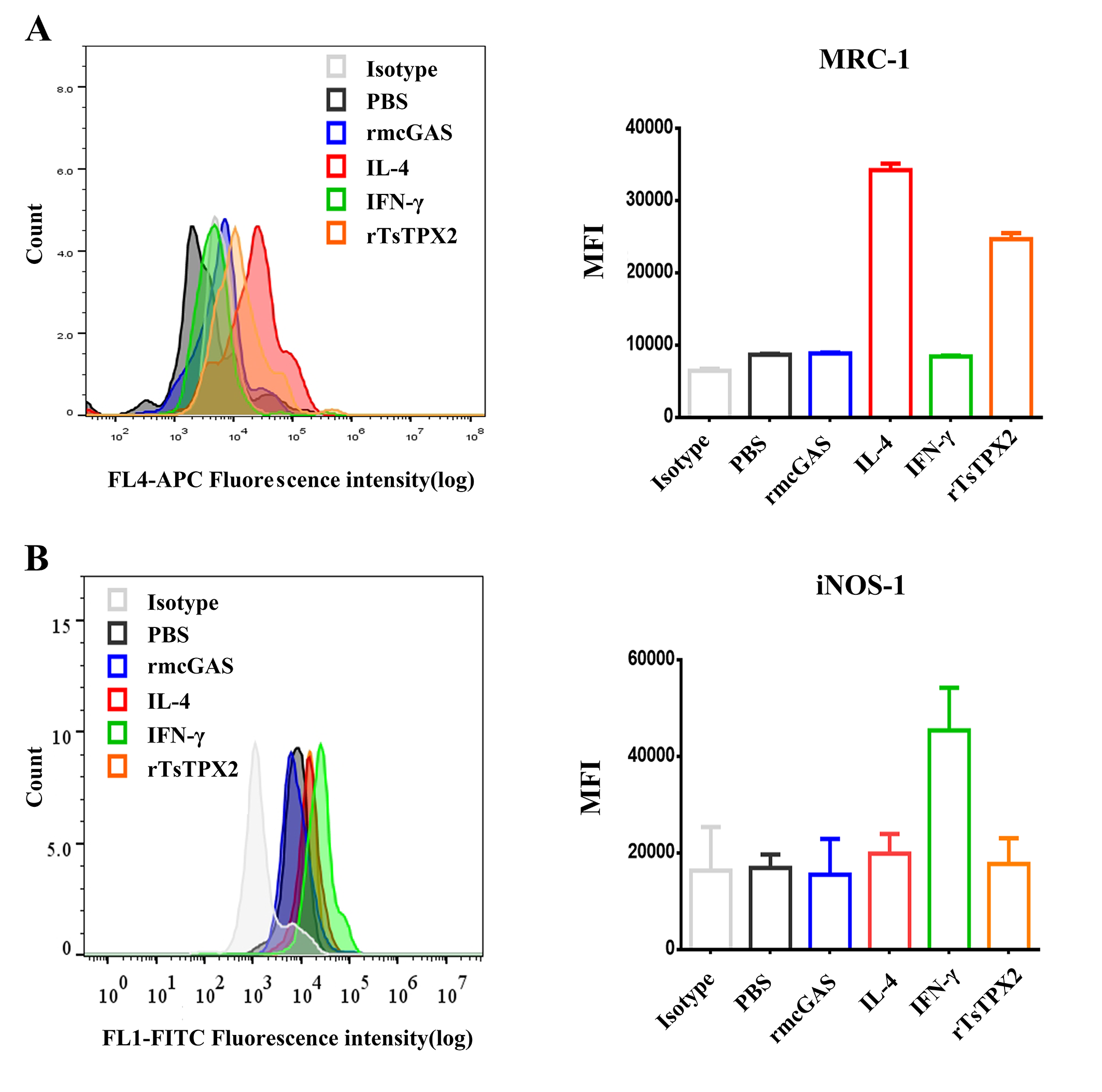

Supplement: Supplementary Figure 4 — The phenotype of macrophages induced in vitro by flow cytometry. The expressions of MRC-1 (A) and iNOS-1 (B). Genes were analyzed by flow cytometry; the left panel was determined by Flowjo software (TreeStar, Ashland, OR, United States), while the right panel shows the mean of fluorescence intensity experimental group. RAW264.7 macrophages were incubated with respective proteins for 48 h. The PBS was used as non-treated control; the recombined mouse Cyclic GMP-AMP synthase (rmcGAS) expressed in the same system with rTsTPX2 was used as irrelevant control; the recombined IL-4 as M2 positive control and IFN-γ as M1 positive control. The following fluorescence conjunct antibodies, Alexa Fluor® 647 rat anti-mouse CD206 (MRC-1), FITC mouse anti-iNOS-1, PE rat anti-mouse F4/80, and the Isotype control antibodies were purchased from BD Biosciences (United States). After staining, the macrophages were firstly gated with F4/80-positive cells, then the iNOS-1 and CD206 (MRC-1) positive cells analyzed with mean of fluorescence intensity (MFI). [file Image_4.TIF]
